# Multiparametric MRI-based nomograms in predicting positive surgical margins of prostate cancer after laparoscopic radical prostatectomy

**DOI:** 10.3389/fonc.2022.973285

**Published:** 2022-09-12

**Authors:** Shuang Meng, Lihua Chen, Qinhe Zhang, Nan Wang, Ailian Liu

**Affiliations:** Department of Radiological, First Affiliated Hospital of Dalian Medical University, Dalian, China

**Keywords:** prostate cancer, positive surgical margins, MRI, nomogram, laparoscopic radical prostatectomy

## Abstract

**Background:**

Positive surgical margins (PSMs) are an independent risk factor of biochemical recurrence in patients with prostate cancer (PCa) after laparoscopic radical prostatectomy; however, limited MRI-based predictive tools are available. This study aimed to develop a novel nomogram combining clinical and multiparametric MRI (mpMRI) parameters to reduce PSMs by improving surgical planning.

**Methods:**

One hundred and three patients with PCa (55 patients with negative surgical margins [NSMs] and 48 patients with PSMs) were included in this retrospective study. The following parameters were obtained using GE Functool post-processing software: diffusion-weighted imaging (DWI); intravoxel incoherent motion model (IVIM); and diffusion kurtosis imaging (DKI). Patients were divided into different training sets and testing sets for different targets according to a ratio of 7:3. The least absolute shrinkage and selection operator (LASSO) regression algorithm was used to analyze the data set to select the optimal MRI predictors. Preoperatively clinical parameters used to build a clinical nomogram (C-nomogram). Multivariable logistic regression analysis was used to build an MRI nomogram (M-nomogram) by introducing the MRI parameters. Based on the MRI and clinical parameters, build an MRI combined with clinical parameters nomogram (MC-nomogram). Comparisons with the M-nomogram and MC-nomogram were based on discrimination, calibration, and decision curve analysis (DCA). A 3-fold cross-validation method was used to assess the stability of the nomogram.

**Results:**

There was no statistical difference in AUC between the C-nomogram (sensitivity=64%, specificity=65% and AUC=0.683), the M-nomogram (sensitivity=57%, specificity=88% and AUC=0.735) and the MC-nomogram (sensitivity= 64%, specificity=82% and AUC=0.756). The calibration curves of the three nomograms used to predict the risk of PSMs in patients with PCa showed good agreement. The net benefit of the MC-nomogram was higher than the others (range, 0.2-0.7).

**Conclusions:**

The mpMRI-based nomogram can predict PSMs in PCa patients. Although its AUC (0.735) is not statistically different from that of the clinical-based nomogram AUC (0.683). However, mpMRI-based nomogram has higher specificity (88% VS. 63%), model stability, and clinical benefit than clinical-based nomogram. And the predictive ability of mpMRI plus clinical parameters for PSMs is further improved.

## Introduction

Laparoscopic radical prostatectomy (LRP) has been widely used in clinical practice and is currently the main way to treat localized prostate cancer (PCa) (1). Of note, 29.1%-34% of patients who undergo LRP have positive surgical margins (PSMs) (2–4), which is an independent risk factor for biochemical recurrence (BCR) in patients after prostatectomy (5, 6). And the results of a cohort study showed that PSMs poses a substantial financial burden (7). Therefore, it is necessary to predict PSMs so that optimal treatment strategies can be implemented.

In previous studies it was reported that there are some preoperative parameters correlate with PSMs after prostatectomy, including age, clinical stage, free prostate specific antigen (FPSA)/total PSA (TPSA), Gleason score, percent of positive cores (PPC), and extra-prostatic extension (3, 8). To predict the risk of advanced PCa, clinicians also often use staging nomograms, such as D’AMICO or CAPRA (9, 10). However, most studies only included the clinical characteristics, and a lack of knowledge about the predictive value of multiparametric MRI (mpMRI).

An mpMRI is considered a common examination for the diagnosis of PCa; specifically, reduces false-negative biopsies (11) and identify risk factors associated with PSMs (12). An mpMRI mainly consists of T2-weighted imaging (T2WI), diffusion-weighted imaging (DWI), and dynamic contrast-enhanced MRI. DWI reflects the PCa histopathologic tissue composition (13) and has the potential to predict abnormal pathologic features after prostatectomy. Over the last two decades, several advanced DWI models have been developed to improve assessment of PCa, including (14). No systematic research studies have shown if advanced DWI models can help clinicians predict PSMs after LRP are lacking. Therefore, the aim of the present study was to develop a novel nomogram combining clinical and mpMRI parameters to predict PSMs after LRP to guide decision-making.

## Materials and methods

### Study population

This single-center, retrospective study included 1055 consecutive male inpatients who underwent pelvic mpMRI between January 2016 and November 2021.

Participants who met the following inclusion criteria were included in our study: ① confirmed diagnosis of PCa by systemic biopsies; ② patients with complete clinical data, including age, Prostate Imaging Reporting and Data System version 2 (PI-RADS v2) score, TPSA, biopsy-based Gleason score, PPC, clinical TNM (cTNM), postoperative Gleason score, pathologic TNM (pTNM), and PSMs locations; and ③ LRP performed on PCa patients by urologists who have performed 500 radical prostatectomies within 3 months after MRI and systemic biopsies. Urologists recommend systematic biopsy of patients before surgery based on PI-RADS and PSA levels. And referring to the mpMRI report to formulate a surgical plan.

Of the participants, those who met at least one of the following criteria were excluded: ① history of PCa treatment; ② incomplete MRI sequences; ③ prostate lesions with poorly-defined boundaries on T2WI and apparent diffusion coefficient (ADC) images, according to PI-RADS v2 (15).

**Figure 1:**
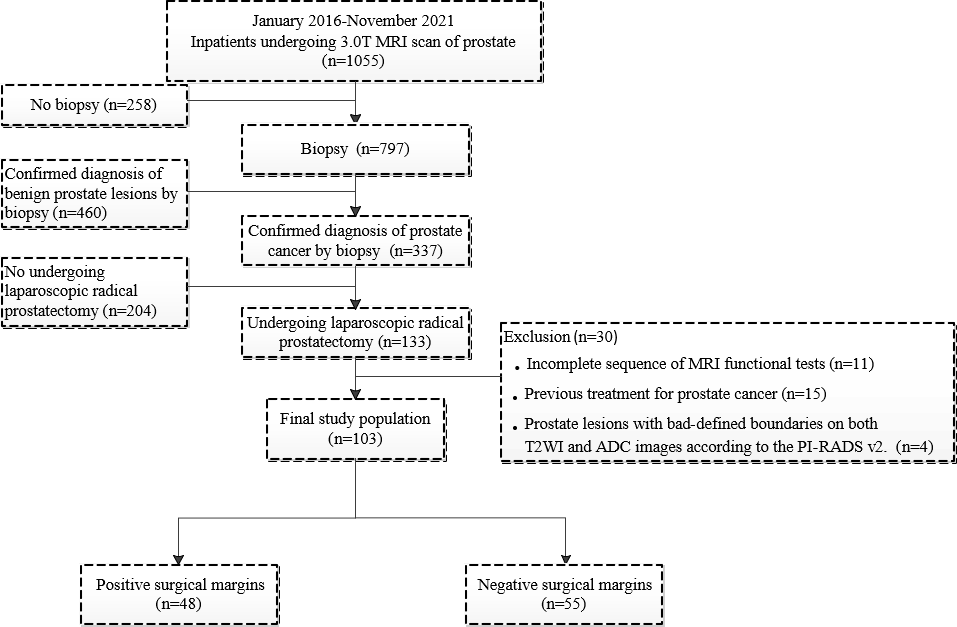
Flowchart of the patient population.

PSMs were defined by cancer cells involving the inked surface of the specimen (16) and divided into negative surgical margins (NSMs) and PSMs groups according to marginal status. One hundred three patients with PCa (55 patients with NSMs and 48 patients with PSMs) were included in our study (**Figure 1**).

The study was approved by the Institutional Review Board of First Affiliated Hospital of Dalian Medical University, informed consent was waived.

### MRI protocols

A 3.0 T MRI scanner (GE-Signa HDXT; GE, Milwaukee, WI, USA) with an eight-channel phased-array body-coil was used in this study. MRI scan was done before the biopsy. The MRI scanning protocol included T1-weighted imaging (T1WI), T2WI, DWI, intravoxel incoherent motion model (IVIM), and diffusion kurtosis imaging (DKI). Sagittal and axial high-definition T2WI sequences were used for prostate tumor location. DWI was performed with high *b* values (up to a maximum of 1000 s/mm^2^). IVIM with *b* values of 0, 20, 50, 100, 150, 200, 400, 800, 1200, 2000, and 3000 s/mm^2^ was performed with a single-shot echo planar (SS-EPI) sequence. DKI with *b* values of 0 and 1500 s/mm^2^ were performed in the oblique axial plane using a SS-EPI sequence with comparable parameters. The diffusion gradients were applied simultaneously along with 15 orthogonal directions. The MRI scanning parameters (DWI, IVIM, DKI, and T2WI) are shown in **Table 1**, supporting information. The images were transferred to an AW 4.4 workstation (GE Healthcare) and reconstructed using GE Functool post-processing software.

**Table 1:** MR sequences parameters of DWI, IVIM, DKI and T2WI.

Parameter	DWI	IVIM	DKI	Axial T2WI	Coronal T2WI	Sagittal T2WI
Pulse sequence name	EPI	EPI	EPI	FRFSE	FRFSE	FRFSE
TR / TE (ms)	4200 / 95	2800 / 90	2500 / 80	5140 / 139	2460 / 128	2660 / 118
Flip angle (°)	90	90	90	90	90	90
FOV (cm)	30 × 30	35 × 31	35 × 35	30 × 30	30 × 30	30 × 30
Voxel (mm)	1.2 × 1.2	1.4 × 1.4	1.4 × 1.4	0.6 × 0.6	0.6 × 0.6	0.6 × 0.6
Matrix	128 × 128	128 × 128	128 × 128	320 × 224	320 × 224	320 × 224
Slice/Thickness (mm)	4.0 / 1.0	7.0 / 1.0	7.0 / 1.0	4.0 / 1.0	5.0 / 1.0	4.0 / 1.0
ETL	–	–	–	23	18	19
Scan Duration(s)	109	151	178	161	101	101
NEX	8	2	2	4	4	4

For analysis of images obtained with DWI, parameter maps were generated by fitting the following models to the pixel signal intensities at the different b values, as follows.

For the mono-exponential DWI model (17),


$$S_{b}/S_{0}=\;exp\text{ (}-b\;\times \;ADC)$$

where *S_b_* is the mean signal intensity with diffusion gradient b, *S_0_* is the mean signal intensity without a diffusion gradient, the b value of ADC_ME_ is 0 and 1,000 s/mm^2^, and the b value of ADC_BE_ is (0, 20, 50, 100, 150, 200, 400, 800, 1200, 2000, and 3000 s/mm^2^).

For the bi-exponential DWI model (18),


$$S_{b}/S_{0}=(1-f)exp(-bD)+\;f\;exp(-bD*)$$

where *S_b_* represents the mean signal intensity with diffusion gradient b and *S_0_* is the mean signal intensity. When b = 0 s/mm^2^, D (D_mono_, D_Bi_) is the true molecular diffusion coefficient. D* (D*_mono_, D*_Bi_) is the pseudodiffusion coefficient and *f* (*f*_mono_, *f*_Bi_) is the perfusion fraction.

For the stretched exponential DWI model (19),


$$S_{b}/S_{0}=\;exp^{\left[-\;\left(b\cdot DDC\right)\right]\;\alpha }$$

where α represents an anomalous exponential term of the intra-voxel water molecule ranging and DDC represents a mean intra-voxel diffusion coefficient.

For the DKI model (20),


$$1n\text{ (}S_{b})=\;1n\;S_{0}-b•D+\;1/6•b^{2}•D^{2}•K$$

where *S_b_* is the MR signal intensity at the particular b value used, *S_0_* is the MR signal intensity without a diffusion gradient, K (FAk, MK, Ka, Kr) is the apparent diffusion kurtosis, and D (FA, Da, Dr, MD) is the ADC revised for non-Gaussian behavior.

### MRI measurements

**Figure 2:**
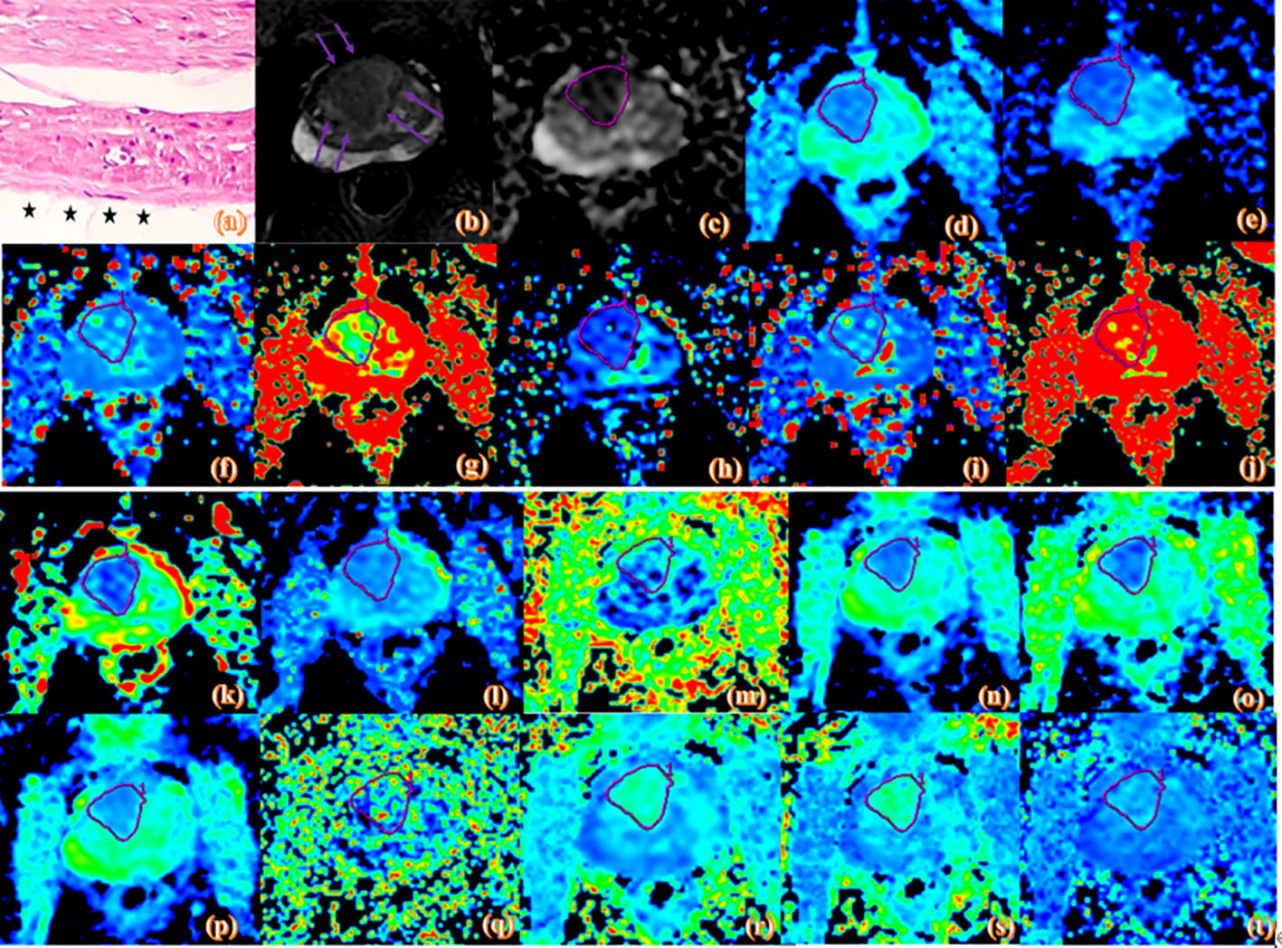
Pathologic and MR parametric maps of a 77-year-old patient (PSA level, 46.4 ng/mL; postoperative Gleason score, 3 + 4 = 7) with negative surgical margins. No neoplastic cells are seen at the ink mark of the margin indicated by the black star on the histologic map (20 × 10 magnification). **(A)**. T2WI map is shown **(B)**, lesion is indicated by pink arrows. ADC map is shown **(C)**, IVIM maps are shown **(D–L)** and DKI maps are shown **(M–T)**. Lesions are indicated by pink ROI. ADC_ME_ value is 0.989×10^-3^mm^2^/s, ADC_BE_ value is 0.716×10^-3^mm^2^/s, D_mono_ value is 0.462×10^-3^mm^2^/s, D*_mono_ value is 0.0046 mm^2^/s, f_mono_ value is 0.426%, D_Bi_ value is 0.417×10^-3^mm^2^/s, D*_Bi_ value is 0.0044 mm^2^/s, f_Bi_ value is 0.486%, DDC value is 0.975×10^-3^mm^2^/s, α value is 0.671, FA value is 0.261, MD value is 1.22 um²/ms, Da value is 1.55 Am²/ms, Dr value is 1.06 um²/ms, FAk value is 0.404, MK value is 0.791, Ka value is 0.91, and Kr value is 0.659.

**Figure 3:**
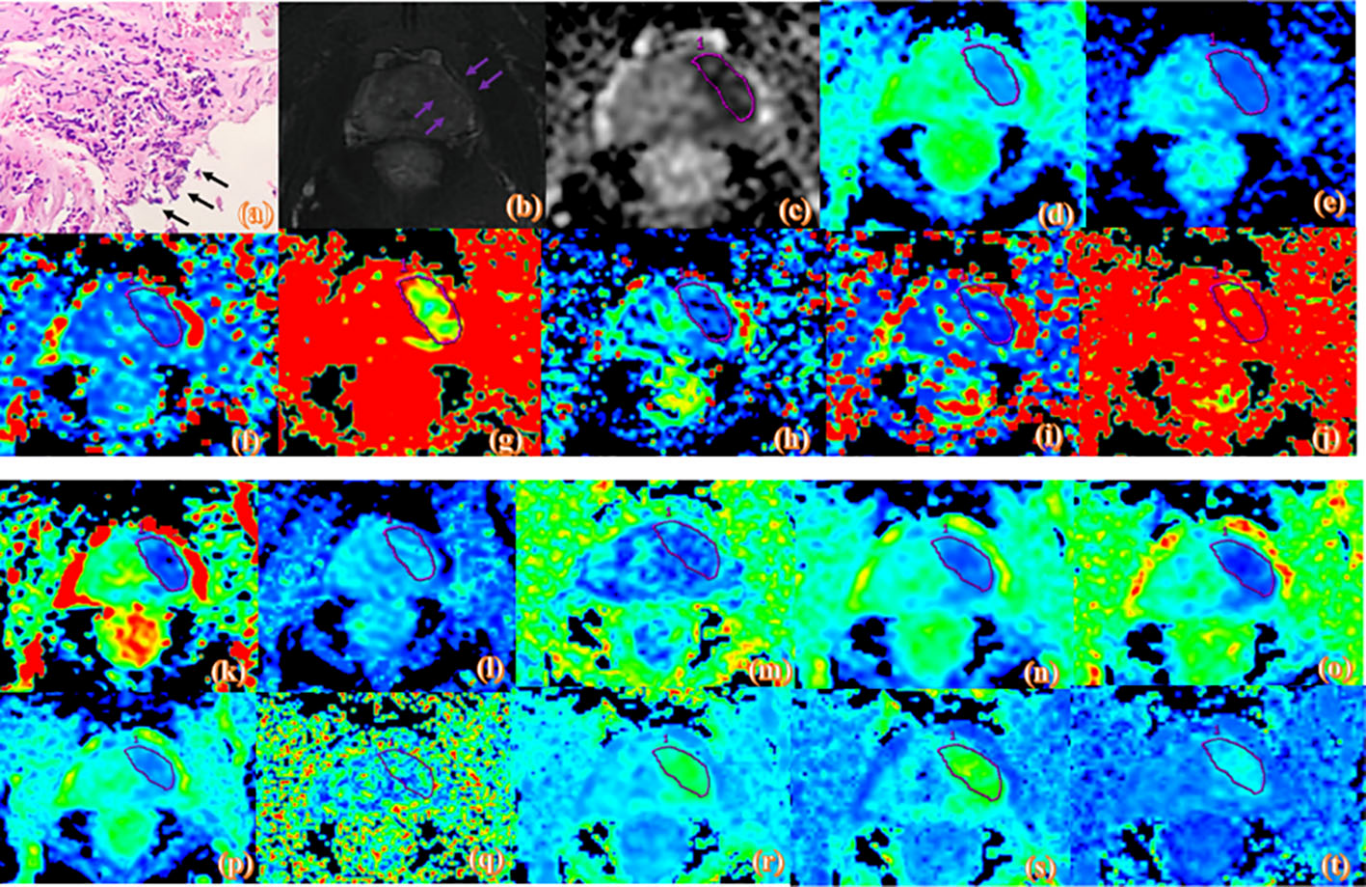
Pathologic and MR parametric maps of a 70-year-old patient (PSA level, 23.2 ng/mL; postoperative Gleason score, 4 + 5 = 9) with positive surgical margins. Neoplastic cells are indicated by black arrows breaking through the edge of the ink blot on the histologic map (20 × 10 magnification). **(A)**. T2WI map is shown **(B)**, lesion is indicated by pink arrows. ADC map is shown **(C)**, IVIM maps are shown **(D–L)** and DKI maps are shown **(M–T)**. Lesions are indicated by pink ROI. ADC_ME_ value is 0.788×10^-3^mm^2^/s, ADC_BE_ value is 0.592×10^-3^mm^2^/s, D_mono_ value is 0.439×10^-3^mm^2^/s, D*_mono_ value is 0.0039mm^2^/s, f_mono_ value is 0.303%, D_Bi_ value is 0.285×10^-3^mm^2^/s, D*_Bi_ value is 0.008 mm^2^/s, f_Bi_ value is 0.476%, DDC value is 0.692×10^-3^mm^2^/s, α value is 0.743, FA value is 0.134, MD value is 1.06 um²/ms, Da value is 1.25 um²/ms, Dr value is 0.963 um²/ms, FAk value is 0.25, MK value is 1.03, Ka value is 1.11, and Kr value is 0.948.

MRI measurements were performed by two experienced radiologists (with 5 and 6 years of experience in abdominal radiology) using the double-blinding method. Prostate MRI interpretation was based on the PI-RADS v2 (15). The region of interest (ROI) was placed by observers in the slice of the largest prostate cancer lesion, and covered the entire lesion while avoiding obvious necrotic or fibrotic areas. The ROI locations on the IVIM and DKI pseudo-color maps were consistent with T2WI and ADC to the greatest extent possible (**Figures 2** and **3**).

### Statistical analysis

All statistical analyses were performed using SPSS (version 25.0; IBM Corp., Armonk, NY, USA), MedCalc (version 15.2.2; Digimizer, Belgium), and R software (version 3.6.1; https://www.R-project.org).

The normality of the data was tested using the Shapiro-Wilk test. Normally distributed continuous variables are expressed as the means ± standard deviations. Non-normally distributed continuous variables are expressed as medians and ranges (25^th^ and 75^th^ percentiles). Nominal variables are expressed as frequencies with percentages.

The differences between two groups were analyzed using two-sided t-tests or the non-parametric Mann-Whitney U test for normally or non-normally distributed data for continuous variables and the Wilcoxon rank-sum test for categorical variables. The inter-observer agreement of the MRI measurements was analyzed by calculating the intraclass correlation coefficient (ICC).

The least absolute shrinkage and selection operator (LASSO) regression algorithm was used to analyze the data set to select the optimal predictors among the mpMRI quantitative parameters. Then, preoperatively clinical parameters used to build a clinical nomogram (C-nomogram), multivariable logistic regression analysis was used to build an MRI nomogram (M-nomogram) and MRI combined with the clinical nomogram (MC-monogram). Further, several kinds of validation methods were used to estimate the accuracy of the nomograms. The receiver operating characteristic (ROC) curve was used to evaluate the discrimination abilities. The area under the curve (AUC) was compared using the DeLong test. The calibration curve was used to evaluate the calibration of the nomogram, and decision curve analysis (DCA) was used to assess the net benefit of nomogram-assisted decisions. A 3-fold cross-validation method was used to randomly split the training cohort into 3 sets, where every two sets were the training sets and the remaining set was the validation set. The average AUC values of the 3 results were used to assess the stability of the nomogram. A two-tailed *P<* 0.05 was considered statistically significant.

## Results

### Participant characteristics

One hundred and three PCa patients with mean age of 71 years (range, 54-83 years) were included in our study. The overall PSMs incidence was 46.6% (48/103). There were significant differences between the two groups with respect to TPSA, PPC and pathological extra-prostatic extension (*P<* 0.05), but not differences in age, PI-RADS v2 score, prostate volume, lesion diameter, biopsy-based Gleason score, cTNM, postoperative Gleason score and pTNM (*P* > 0.05). The clinical characteristics are shown in **Table 2**. A stratified sampling method was used to divide the data into the training set and testing set at a ratio of 7:3. Of the 103 patients in this study, 72 were assigned to the training set, and 31 were assigned to the testing set. There were no significant differences in clinical characteristics between the training and testing sets. The details are shown in **Tables 3** and **4**.

**Table 2:** Clinical characteristics of patients with negative and positive margins.

Characteristics	NSMs (n = 55)	PSMs (n = 48)	*P value*
Age (year), median [IQR]	72 (67–77)	71 (67–76)	0.743
TPSA (ng/ml), median [IQR]	15.53 [10.17-26.35]	25.52 [14.85-56.22]	0.004*
PI-RADS v2, n (%)			0.336
4	54 (98)	45 (94)	
5	1 (2)	3 (6)	
Prostate volume (ml)	34.36(IQR,47.18-23.38)	34.16(IQR,54.05-27.71)	0.248
Lesion diameter (cm)	1.45 (IQR,1.97-1.00)	1.10 (IQR,1.80-0.60)	0.061
Biopsy Gleason score, n (%)			0.133
ISUP 1	18 (33)	7 (15)	
ISUP 2	4 (7)	5 (10)	
ISUP 3	6 (11)	5 (10)	
ISUP 4	19 (35)	16 (33)	
ISUP 5	8 (15)	15 (32)	
Percent of positive cores, median [IQR]	0.33 [0.17-0.58]	0.63 [0.33-0.83]	< 0.001*
cTNM, n (%)			0.280
T2a	47 (85)	39 (81)	
T2b	2 (4)	0	
T2c	6 (11)	9 (19)	
Postop Gleason score, n (%)	PSM	NSM	0.083
ISUP 1	7 (13)	6 (12.5)	
ISUP 2	12 (22)	6 (12.5)	
ISUP 3	4 (7)	11 (23)	
ISUP 4	10 (18)	13 (27)	
ISUP 5	22 (40)	12 (25)	
pTNM, n (%)			0.334
pT2	43 (78)	39 (81)	
pT3a	3 (6)	0	
pT3b	9 (16)	9 (19)	
Pathological extra-prostatic extension			0.001*
No	30 (62.5)	52 (94.5)	
Yes	18 (37.5)	3 (5.5)	
Positive margin position, n (%)			—
Peripheral margin	—	31	
Tip incisal margin	—	19	
Basal margin	—	21	

NSMs, negative surgical margins; PSMs, positive surgical margins, TPSA, total prostate specific antigen. *P value is statistically significant.

**Table 3:** Clinical characteristics of the training and testing sets for MRI nomogram predicting PSMs.

Variable	Training set	Testing set	*p*-value
NSMs	38 (52.8)	17 (54.8)	0.847
PSMs	34 (47.2)	14 (45.2)	
Age, y	71 ± 7	71 ± 6	0.816
TPSA, ng/ml	17.7 (IQR,44.3-11.2)	19.1 (IQR,44.9-14.0)	0.326
Biopsy Gleason score, n (%)			0.542
ISUP 1	17 (23.6)	8 (25.8)	
ISUP 2	5 (6.9)	4 (12.9)	
ISUP 3	7 (9.7)	4 (12.9)	
ISUP 4	24 (33.3)	11 (35.5)	
ISUP 5	19 (26.4)	4 (12.9)	
Percent of positive cores, median [IQR]	0.46 (IQR,0.81-0.17)	0.42 (IQR,0.67-0.17)	0.610
Postop Gleason score, n (%)			0.463
ISUP 1	9 (12.5)	4 (12.9)	
ISUP 2	12 (16.7)	6 (19.4)	
ISUP 3	11 (15.3)	4 (12.9)	
ISUP 4	13 (18.1)	10 (32.3)	
ISUP 5	27 (37.5)	7 (22.6)	
pTNM, n (%)			0.196
pT2	54 (75.0)	28 (90.3)	
pT3a	3 (4.2)	0 (0.0)	
pT3b	15 (20.8)	3 (9.7)	
cTNM, n (%)			0.210
T2a	58 (80.6)	28 (90.3)	
T2b	2J (2.8)	0 (0.0)	
T2c	12 (16.7)	3 (9.7)	
PI-RADS			1.000
4	69 (95.8)	30 (96.8)	
5	3 (4.2)	1 (3.2)	

NSMs, negative surgical margins; PSMs, positive surgical margins, TPSA, total prostate specific antigen.

**Table 4:** Clinical characteristics of the training and testing sets for MRI combined with clinical parameters nomogram predicting PSMs.

Variable	Training set	Testing set	*p*-value
NSMs	38 (53)	17 (55)	0.847
PSMs	34 (47)	14 (45)	
Age, y	72 ± 6	70 ± 7	0.248
TPSA, ng/ml	19.4 (IQR,53.1-12.5)	17.3 (IQR,30.6-8.5)	0.052
Biopsy Gleason score, n (%)			0.602
ISUP 1	17 (23.6)	8 (25.8)	
ISUP 2	6 (8.3)	3 (9.7)	
ISUP 3	8 (11.1)	3 (9.7)	
ISUP 4	22(30.6)	13(41.9)	
ISUP 5	19(26.4)	4(12.9)	
Percent of positive cores, median [IQR]	0.50 (IQR,0.83-0.19)	0.42 (IQR=0.58-0.17)	0.178
Postop Gleason score, n (%)			0.542
ISUP 1	9 (12.5)	4 (12.9)	
ISUP 2	11 (15.3)	7 (22.6)	
ISUP 3	11 (15.3)	4 (12.9)	
ISUP 4	14 (19.4)	9 (29.0)	
ISUP 5	27 (37.5)	7 (22.6)	
pTNM, n (%)			0.068
pT2	53 (73.6)	29 (93.5)	
pT3a	3 (4.2)	0 (0.0)	
pT3b	16 (22.2)	2 (6.5)	
cTNM, n (%)			0.889
T2a	60 (83.3)	26 (83.9)	
T2b	2 (2.8)	0 (0.0)	
T2c	10 (13.9)	5 (16.1)	
PI-RADS			1.000
4	69 (95.8)	30 (96.8)	
5	3 (4.2)	1 (3.2)	

NSMs, negative surgical margins; PSMs, positive surgical margins, TPSA, total prostate specific antigen.

### Consistency analysis

As shown in **Table 5**, supporting information, the ICC values were > 0.9, which suggested excellent inter-observer agreement.

**Table 5:** Two-observer measurement consistency.

Variable	NSMs (n = 55)			PSMs (n = 48)		
	Observer 1	Observer 2	ICC	Observer 1	Observer 2	ICC
ADC_ME_ (×10^-3^mm^2^/s)	0.943 ± 0.03	0.941 ± 0.028	0.993	0.876 ± 0.021	0.875 ± 0.0209	0.993
IVIM						
ADC_BE_	0.725 ± 0.019	0.723 ± 0.02	0.993	0.636 ± 0.014	0.638 ± 0.014	0.99
D_mono_ (×10^-3^mm^2^/s)	0.517 ± 0.014	0.517 ± 0.015	0.991	0.455 ± 0.009	0.457 ± 0.009	0.984
D*_mono_ (mm^2^/s)	0.0192 ± 0.006	0.0187 ± 0.006	0.998	0.01 ± 0.003	0.009 ± 0.002	0.993
*f*_mono_ (%)	0.385 ± 0.013	0.384 ± 0.013	0.992	0.338 ± 0.009	0.337 ± 0.009	0.973
D_Bi_ (×10^-3^mm^2^/s)	0.478 ± 0.025	0.479 ± 0.026	0.985	0.343 ± 0.02	0.37 ± 0.027	0.875
D*_Bi_ (mm^2^/s)	0.0254 ± 0.007	0.0248 ± 0.007	0.997	0.0183 ± 0.004	0.0178 ± 0.004	0.996
*f*_Bi_ (%)	0.444 ± 0.018	0.448 ± 0.019	0.991	0.436 ± 0.017	0.437 ± 0.017	0.985
DDC (×10^-3^mm^2^/s)	0.966 ± 0.043	0.947 ± 0.046	0.96	0.754 ± 0.032	0.762 ± 0.034	0.982
α	0.705 ± 0.012	0.71 ± 0.013	0.99	0.706 ± 0.012	0.705 ± 0.011	0.995
DKI						
FA	0.214 ± 0.009	0.214 ± 0.008	0.993	0.213 ± 0.008	0.213 ± 0.008	0.996
MD (um²/ms)	1.171 ± 0.029	1.177 ± 0.03	0.972	0.415 ± 0.131	0.414 ± 0.137	0.996
Da (um^2^/ms)	1.426 ± 0.035	1.43 ± 0.036	0.972	0.415 ± 0.131	0.414 ± 0.137	0.996
Dr (um^2^/ms)	1.03 ± 0.029	1.43 ± 0.036	0.972	0.415 ± 0.131	0.414 ± 0.137	0.996
FAk	0.30 ± 0.013	0.30 ± 0.013	0.992	0.31 ± 0.015	0.31 ± 0.014	0.985
MK	0.904 ± 0.021	0.904 ± 0.021	0.987	0.969 ± 0.024	0.969 ± 0.024	0.991
Ka	0.973 ± 0.023	0.971 ± 0.023	0.994	1.081 ± 0.03	1.079 ± 0.031	0.989
Kr	0.809 ± 0.02	0.809 ± 0.02	0.993	0.87 ± 0.022	0.868 ± 0.021	0.992

NSMs, negative surgical margins; PSMs, positive surgical margins.

### Correlations between mpMRI parameters and PSMs

**Figure 4:**
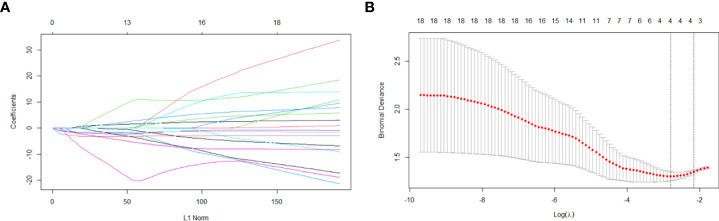
Variable selection based on the LASSO regression model. A coefficient profile plot was produced against the log(lambda) sequence **(A)**. Four variables with non-zero coefficients were selected by optimal lambda. By verifying the optimal parameter (lambda) in the LASSO model, the partial likelihood deviance (binomial deviance) curve was plotted versus log(lambda) and dotted vertical lines were drawn based on 1 standard error criterion **(B)**.

There were four potential predictors selected on the basis of the data from the 103 patients with non-zero coefficients in the LASSO regression model, including Da, D_mono_, D_Bi_, and DDC (**Figure 4**).

### Prediction model development

**Figure 5:**
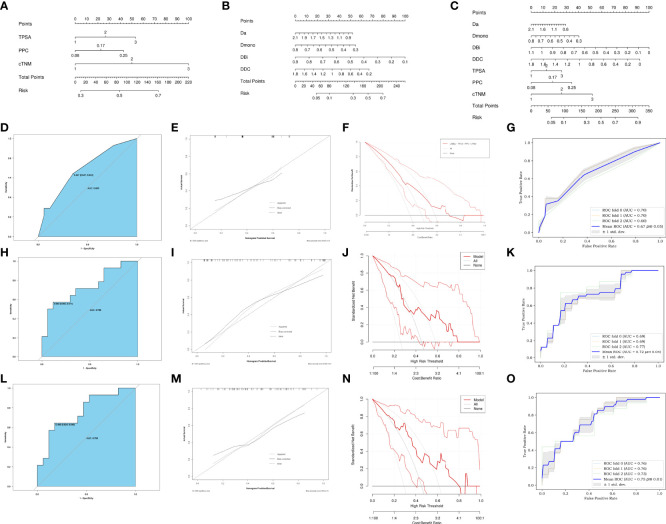
Development of C-nomogram **(A)**, M-nomogram **(B)** and MC-nomogram **(C)** predicting PSMs after laparoscopic radical prostatectomy. **(D, H, L)** ROC validation of the PSMs risk nomogram prediction. The blue area represented the performance of the nomogram. **(E, I, M)** Calibration curves of the PSMs risk nomogram prediction. The y-axis represents the actual diagnosed PSMs. The x-axis represents the predicted risk of PSMs. The diagonal dotted line represents a perfect prediction by an ideal model. The solid line represented the performance of the C-nomogram **(D)**, M-nomogram **(H)** and MC-nomogram **(L)**, which indicated that a closer fit to the diagonal dotted line represented a better prediction. **(F, J, N)** Decision curve of the PSMs risk nomogram prediction. The y axis represents the net benefit and the x axis represents the risk threshold. The thick solid line represents the assumption that all patients had no PSMs. The thin solid line represents the assumption that all patients had PSMs. The red line represents the risk nomogram. **(F)** From C-nomogram, **(J)** the M-nomogram and **(N)** MC-nomogram. The net benefit of the MC-nomogram is highest in the range from 0.2-0.7.). **(G, K, O)** Mean ROC curve of the nomogram to predict PSMs after 3-fold cross-validation. TPSA, total prostate specific antigen; PPC, percent of positive cores.

Introducing preoperatively clinical parameters TPSA, PPC, and cTNM as independent predictors, a C-nomogram was developed and is presented in **Figure 5A**. Introducing the Da, D_mono_, D_Bi_, and DDC as independent predictors, an M-nomogram was developed and is presented in **Figure 5B**. Introducing the MRI and preoperatively clinical parameters as independent predictors, an MC-nomogram was developed and is presented in **Figure 5C**.

### Comparison of predictive model performance

The ROC of the C-nomogram (sensitivity=64%, specificity=65% and AUC=0.683), the M-nomogram (sensitivity=57%, specificity=88% and AUC=0.735) and the MC-nomogram (sensitivity= 64%, specificity=82% and AUC=0.756) were shown in **Figures 5D, H, L**. The models had moderately good performance. Although there was no statistical difference in AUC between the three models (C-nomogram VS. M-nomogram, *p*=0.71, C-nomogram VS. CM-nomogram, *p*=0.05, M-nomogram VS. CM-nomogram, *p*=0.1). However, the specificity of M-nomogram was better than that of C-nomogram, and the model was optimized after MRI parameters was combined with clinical parameters, and the optimal sensitivity and specificity were obtained (sensitivity= 64%, specificity=82%).

The calibration curves of the three nomograms used to predict the risk of PSMs in patients with PCa also showed good agreement (**Figures 5E, I, M**).

The decision curve analysis for the PSMs risk nomograms were presented in (**Figures 5F, J, N**). The decision curve showed that it would be more accurate to use MC-nomogram in the current study to predict the risk of PSMs in the range from 0.2 - 0.7.

The 3-fold cross-validation of the nomograms demonstrated its stability in predicting PSMs (**Figures 5G, K, O**).

## Discussion

PSMs in PCa patients are commonly associated with BCR and a higher risk for secondary treatment (21, 22). Therefore, identifying predictive factors may help urologists select the at-risk patients who are more likely to benefit from LRP therapy and the optimal surgical procedure can be planned. For high-risk patients, who can choose to retain one side or not to retain the neurovascular bundle during surgery, or to combine ADT before surgery to reduce the rate of PSMs. Previous studies have shown that several clinical and pathologic factors have the potential of predicting PSMs (4, 8, 23). Zhang et al. (8) conducted a comprehensive meta-analysis and systematic review with a sample of 50,014 patients, and showed that TPSA, biopsy-based Gleason score, postoperative Gleason score, pTNM, positive lymph nodes, extra-prostatic extension, and seminal vesicle invasion are independent prognostic factors for PSMs. None of the studies, however, have systematically predicted the post-LRP margin status based on mpMRI. Currently, mpMRI is widely recommended for detection and localization of PCa, and studies have indicated that mpMRI improves the predictions of preoperative clinical nomograms (24, 25). And the application of artificial intelligence (26) and mpMRI-3D model (27) also provides more possibilities to reduce the rate of PSMs. Herein, we developed three nomograms based on clinical, mpMRI and mpMRI combined with clinical parameters. Further, the performance of the three prediction models was compared.

First, the PSMs rate was 46.6%, which was higher than the results reported by Qu et al. (4) (PSMs rate = 34%). The reason for the difference may be that the patients in our study were diagnosed at a later stage; specifically, 56.3% of the patients had a biopsy-based Gleason score ≥4 compared to 38% of patients in their study.

Moreover, we showed that Da, D_mono_, D_Bi_, and DDC were associated with PSMs risk in PCa patients based on LASSO regression analysis. The M-nomogram achieved a higher AUC (0.735) compared to C-nomogram (0.683) for PSMs prediction, and suggested that lower Da, Dmono, DBi, and DDC were the key parameters that determined the risk of PSMs for PCa patients. Because as tumors grow, both cell overcrowding and changes in stroma production alter cell-stroma and cell-cell associations in an ongoing dynamic process that disturbs the microarchitecture (28). These microstructural changes promote the proliferation of tumor cells and the interstitial transition of tumor cells, resulting in restricted diffusion of water molecules. Moreover, Da, D_mono_, D_Bi_ and DDC reflect the diffusion of water molecules in and out of cells. Therefore, the decrease in Da, D_mono_, D_Bi_, and DDC values in the PSMs group may be related to an increase in cell number and the loss of interstitial matrix. This is consistent with the previous parameters of Alessi et al. (29), who also showed that ADC performs well in PSMs prediction, with lower ADC values observed in PSMs patients. The ADC model, however, tends to oversimplify the complexity of prostate tissue while ignoring the biological specificity of PCa, which results in poor predictive performance (30). Bourne et al. (31) compared the information content of four phenomenologic diffusion models in whole prostate tissue *ex-vivo* using the Akaike information criterion. Bourne et al. (31) found the biexponential and DKI model to have a higher information content than the mono-exponential DWI model. Therefore, we attempted to construct a preoperative prediction model of PSMs using a more complex model that might provide a richer informative description of DWI signals in PCa and obtain better predictive power.

Recent studies have also found that preoperative mpMRI can be used to predict PSMs and appears to have a significant favorable impact on surgical planning. A retrospective study of 179 patients with in-house robotic assisted LRP, M. Quentin et al. found that length of capsular tumor contact was the best MRI predictor for PSMs at the capsule and distance to the membranous urethra for tumors with PSMs at the apical urethra (32). Irini Youssef et al. found that pathologic T-stage, anteroposterior pelvic outlet and pelvic depth were risk factors for positive margins (33). The findings were consistent with our study. However, our study highlights the application of functional sequences. We combine different DWI models to screen for optimal functional parameters. The focus is on the predictive power of the biological behavior of the tumors themselves for positive margins, which has rarely been addressed in previous studies.

In addition, by incorporating preoperative clinical indicators, a combined risk MC-nomogram was created. The MC-nomogram also had a higher AUC (0.756). Comparison of the calibration curve and DCA, showed that MC-nomogram demonstrated relatively good calibration power and clinical net benefit. This finding indicates that the ability of MRI-based nomogram to predict PSMs was optimized after combining clinical indicators. He et al. (34) reported that radiomics signatures based on ADC predict PSMs (AUC=0.733), and when combined with clinical parameters, improves the model efficiency (AUC=0.766). This finding is consistent with our parameters. Preoperative mpMRI can be used to predict PSMs and appears to have a significant favorable impact on surgical planning (35, 36). However, previous studies mostly used mpMRI to predict pathologic extra-prostatic extension (37), and there were limited studies to predict PSMs. Model performance was improved when clinical parameters were included in the mpMRI prediction model. A recent study showed that the nomogram described by Gandaglia et al. (38) using an MRI combined with clinical parameters as a staging method improved discrimination in predicting postoperative adverse pathologic factors (38). Therefore, it is difficult to predict PSMs only using preoperative mpMRI or clinical parameters, and a combination of the them is necessary.

Our study had some significant limitations. First, this was a single-center retrospective study, and thus, the limited sample size may cause selection bias and other confounding factors. Second, the MC-nomogram lacked external validation. Third, we constructed predictive models based on mpMRI parameters obtained from complex DWI models, which may have limited the application and multicenter generalization of the MC-nomogram. And more our study did not include robot-assisted LRP, so results could be not applicable in robotic-assisted LRP.

## Conclusions

The mpMRI-based nomogram can predict PSMs in PCa patients. Although its AUC (0.735) is not statistically different from that of the clinical-based nomogram AUC (0.683). However, mpMRI-based nomogram has higher specificity (88% VS. 63%), model stability, and clinical benefit than clinical-based nomogram. And the predictive ability of mpMRI plus clinical parameters for PSMs is further improved.

## Data availability statement

The raw data supporting the conclusions of this article will be made available by the authors, without undue reservation.

## Ethics statement

The studies involving human participants were reviewed and approved by the institutional review board of First Affiliated Hospital of Dalian Medical University. Written informed consent for participation was not required for this study in accordance with the national legislation and the institutional requirements.

## Author contributions

SM and AL conceived of the presented idea. SM and LC performed the measurements. AL supervised the work. SM and QZ processed the experimental data and performed the analysis. SM drafted the manuscript and NW aided in working on the manuscript. All authors contributed to the article and approved the submitted version.

## Conflict of interest

The authors declare that the research was conducted in the absence of any commercial or financial relationships that could be construed as a potential conflict of interest.

## Publisher’s note

All claims expressed in this article are solely those of the authors and do not necessarily represent those of their affiliated organizations, or those of the publisher, the editors and the reviewers. Any product that may be evaluated in this article, or claim that may be made by its manufacturer, is not guaranteed or endorsed by the publisher.

## Abbreviations

ADC, apparent diffusion coefficient; ADC_ME_, mono-exponential apparent diffusion coefficient; ADC_BE_, bi-exponential apparent diffusion coefficient; AUC, area under the curve; BCR, biochemical recurrence; cTNM, clinical TNM; Da, axial diffusion; Dr, radial diffusion; DCA, decision curve analysis; DDC, mean intra-voxel diffusion coefficient; DKI, diffusion kurtosis imaging; DWI, diffusion-weighted imaging; FA, fractional anisotropy; FAK, fractional anisotropy kurtosis; FPSA, free prostate specific antigen; ICC, intraclass correlation coefficient; IVIM, intravoxel incoherent motion model; Ka, axial kurtosis; Kr, radial kurtosis; LASSO, least absolute shrinkage and selection operator; LRP, laparoscopic radical prostatectomy; M-nomogram, MRI nomogram; MC-monogram, MRI combined with the clinical nomogram; MD, mean diffusion; MK, mean kurtosis; mpMRI, multiparametric MRI; NSM, negative surgical margin; PCa, prostate cancer; PPC, percent of positive cores; PSMs, positive surgical margins; pTNM, pathologic TNM; ROC, receiver operating characteristic curve; SS-EPI, single-shot echo planar; T1WI, T1-weighted imaging; T2WI, T2-weighted imaging; TPSA, total prostate specific antigen1

## Glossary

**Table:**

ADC	apparent diffusion coefficient
ADCME	Monoexponential apparent diffusion coefficient
ADCBE	bi-exponential apparent diffusion coefficient
AUC	area under the curve
BCR	biochemical recurrence
cTNM	clinical TNM
Da	axial diffusion
Dr	radial diffusion
DCA	decision curve analysis
DDC	mean intra-voxel diffusion coefficient
DKI	diffusion kurtosis imaging
DWI	diffusion-weighted imaging
FA	fractional anisotropy
FAK	fractional anisotropy kurtosis
FPSA	free prostate specific antigen
ICC	intraclass correlation coefficient
IVIM	intravoxel incoherent motion model
Ka	axial kurtosis
Kr	radial kurtosis
LASSO	least absolute shrinkage and selection operator
LRP	laparoscopic radical prostatectomy
M-nomogram	MRI nomogram
MC-monogram	MRI combined with the clinical nomogram
MD	mean diffusion
MK	mean kurtosis
mpMRI	multiparametric MRI
NSM	negative surgical margin
PCa	prostate cancer
PPC	percent of positive cores
PSMs	positive surgical margins
pTNM	pathologic TNM
ROC	receiver operating characteristic curve
SS-EPI	single-shot echo planar
T1WI	T1-weighted imaging
T2WI	T2-weighted imaging
TPSA	total prostate specific antigen1
